# Multiple sclerosis outpatient future groups: improving the quality of participant interaction and ideation tools within service improvement activities

**DOI:** 10.1186/s12913-015-0773-8

**Published:** 2015-04-23

**Authors:** Alison Thomson, Carol Rivas, Gavin Giovannoni

**Affiliations:** Centre for Neuroscience and Trauma, Blizard Institute, Queen Mary, University of London, Whitechapel, London E1 2AT, UK; Centre for Primary Care and Public Health Blizard Institute, Barts and The London School of Medicine and Dentistry, Yvonne Carter Building, 58 Turner Street, London, E1 2AB UK; School of 717 Health Sciences, University of Southampton, Highfield, Building 67, 718, Southampton, SO17 1BJ UK

**Keywords:** Multiple sclerosis, Design, Props, Patient experience, Outpatients, Focus group, Quality

## Abstract

**Background:**

Improving the patient experience is a key focus within the National Health Service. This has led us to consider how health services are experienced, from both staff and patient perspectives. Novel service improvement activities bring staff and patients together to use design-led methods to improve how health services are delivered. The Multiple Sclerosis Outpatient Future Group study aimed to explore how analogies and props can be used to facilitate rich interactions between staff and patients within these activities. This paper will consider how these interactions supported participants to share experiences, generate ideas and suggest service improvements.

**Method:**

Qualitative explorative study using ‘future groups,’ a reinterpretation of the recognised focus groups method directed towards exploring future alternatives through employing analogies and physical props to engage participants to speculate about future service interactions and health experiences. Participants were people with multiple sclerosis (PwMS) and outpatient staff: staff nurses, nursing assistants, junior sisters and reception staff.

**Results:**

Use of future groups, analogies and physical props enabled PwMS and outpatient staff to invest their own ideas and feelings in the service improvement activity and envisage alternative health care scenarios. The combination of participants in the groups with their diverse perspectives and knowledge of the service led to a collaborative approach in which staff highlighted potential practical problems and patients ensured ideas were holistic. Service improvements were prototyped and tested in the outpatient clinic.

**Conclusion:**

Design-led methods such as future groups using analogies and physical props can be used to facilitate interactions between staff and patients in service improvement activities, leading to the generation of meaningful ideas. It is hoped that improving the quality of ideation tools within design-led methods can contribute to developing successful service interventions in service improvement activities.

## Background

Improving the patient experience through service redesign and greater information provision has been a key concern within health policy for a few years [1,2]. It is now widely acknowledged that the active engagement of front line staff is necessary to improve the quality of interactions and conversations between staff and patients [3,4]. Health professionals and researchers have responded by working with professional designers to involve patients and staff in service design activities. These projects use design-led, participatory approaches to encourage non-designers to think differently about how health services could be delivered. Experience Based Co-design (EBCD) is such an approach and can be implemented with the support of a free-to-access online toolkit [5,6]. As an emerging social practice [7], co-design allows people who do not consider themselves designers to carry out the design activity using design tools to assist them. In EBCD, areas in the care pathway are identified where patient and staff experiences are critical as it is believed that patients can effectively work together with staff to redesign these situations using this approach. The attraction of the EBCD toolkit for the NHS is that it is available for health care teams to conduct within their services and does not need external leadership or the involvement of professional designers [8].

A review of the EBCD approach reported its development since conception in 2005. It stated that EBCD is at the core of over 80 projects in seven different countries, some completed, some ongoing and others planned [9]. However the long term benefits of EBCD activities are unknown and the report itself, alongside others, acknowledges the need for more robust studies of the effectiveness of this approach [9,10]. Another review of service design projects and their long-term impacts similarly noted that although these techniques are seen as successful in the service improvement discipline there was a lack of formal evaluation of the method’s success in improving health related outcomes [10].

Within EBCD, the co-design activity itself was said to be the most challenging element that reported projects struggled with [9]. Tools and techniques employed within the original toolkit when the approach was developed included filming patients and staff, service diagrams showing the patient journey, post-it notes, prototyping and scenario building [5]. In King’s College London’s Report evaluating the development of EBCD, out of 47 respondents to the question regarding whether the completed projects had used the King’s Fund online toolkit, only 19 % of projects used it extensively, 26 % used bits of it, with 20 % either not knowing of it or not using it [9].

Although the approach is increasing in use [9], little is known about how the EBCD toolkit is used in practice and whether the toolkit approach is successful as a method for delivering design-led activities amongst non-designers [11]. It has been described that there is the potential for the creative traits of design to be harnessed by non-designers in these collaborative activities but that this requires effective resources to be able to achieve this [12]. The range of ideation tools within EBCD have been reviewed as limited in that they do not suppport participants to go beyond suggesting simple solutions to immediate issues [8]. This causes concern as more radical solutions and possibilities for new service innovations are not explored. This suggests a need for better tools to be used within these activities to assist in the design activity.

There are also fundamental tensions between co-design’s intended aims and its actual practice [9]. The core principle of co-design is based on an equal partnership between staff and patients to enable them to share their experiences and create solutions to problems, although several issues have emerged around implementing solutions within existing organizational structures. For example, the undoubtable strength of the participatory approach is around sharing stories, building trust and finding a common ground between participants, yet this form of democratic decision making could potentially be problematic when the approach is led by health care managers. Within the co-design session, handing over some control and power to patients is novel but it is unclear how this power is, or should be, re-negotiated when the patients are at their next appointment and staff return to their daily roles [7].

This paper reports on the MS Outpatient Future Group study which demonstrates how, through working with a Design Researcher, speculation through analogies can be used as a design tool by non-designers in a service improvement project. Using ideation tools, the approach can explore new health service possibilities by helping participants to envisage alternative service experiences. The notion of speculating to imagine alternative futures is influenced by the speculative design discipline [13]. In this specific field, speculation is used to imagine alternative ways of living for the purpose of reflecting and developing discussions on current issues. This study differs from EBCD insofar as the Design Researcher developed bespoke ideation tools for use in the study. This study also contributes to participatory health services research literature by exploring the potential of new ideation tools to facilitate staff and patient interaction. It considers how the role of a professional designer as the facilitator can develop more radical ideas, beyond solving simple problems, from patients and staff in a service improvement activity.

## Methods

### Setting

The MS Outpatient Future Group study was developed by a Design Researcher working with a Professor of Neurology. The research team consisted of a Design Researcher (AT), Medical Sociologist (CR), Matron of Outpatients (TD) and Professor of Neurology (GG) at Queen Mary, University of London and the Royal London Hospital (RLH). The study received National Research Ethics Service ethical approval (12/LO/1098, 3rd September 2012) and was conducted over three months.

The outpatient clinic was chosen as it is a key access point for relevant medical knowledge and specialist services for PwMS. It is in the outpatient clinic where PwMS have regular consultations with their Neurologist and MS Specialist Nurse, get access to disease modifying therapies and receive access to wider care services. Previous to this study, the outpatient staff were involved in a project entitled ‘Big Brother diary room’ in which video diaries of patients were shown to staff. This probably contributed to the willingness of staff to take part in the Futures Group study.

### Recruitment

Patients were initially approached in the clinic by a member of the clinical team, before meeting the Design Researcher. The patient sample was purposefully selected from the MS outpatient clinic at RLH as patient participants had to have had experience of being treated in this clinic. Two male and three female patients were successfully recruited alongside eight female members of staff. The sampling procedure for the outpatient staff group was naturally occurring [14], i.e. the nurses were already working together in the MS clinic. The outpatient management team arranged the opportunity for outpatient staff to be relieved of their nursing duties for a morning and an afternoon to take part in the study. Full written informed consent was given by all participants.

The Design Researcher developed a visual identity for the study (logo and colour palette) to be applied to all the printed material. The purpose of this was to differentiate the study from other research studies that the patients or staff may have previously had experience with. The resulting paperwork was significantly different than standard NHS stationery as it conveyed an inviting aesthetic.

### Future group procedure

The study explored how focus groups can elicit feedback and encourage discussions between participants [14,15]. Although the interaction aspect of the methodology is pivotal [16,17] and open to analysis [18] there have been few attempts to examine the impact of these interactions on the focus group participants or outcomes [19]. This study considers the potential for focus groups as a method to be reimagined as ‘future groups’ to bring participants together to share and acquire knowledge from other participants while also co-constructing ideas for the future.

**Figure 1:**
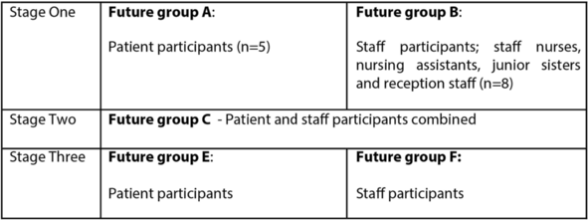
**Future group procedure.**

There were three stages to the study, each stage consisting of two future group sessions. Each participant attended three future groups, one in each stage. Figure 1 displays how these worked. Every future group session was sound recorded, then transcribed by the Design Researcher and analysed using NVivo Version 8. Data was extracted and for the work reported in this paper, themes were informed by using Grounded Theory principles [20]. The Design Researcher facilitated the discussions within the group, with the Medical Sociologist as non-participant observer, taking field notes.

### Stage 1 – mapping the staff and patient journey

In the first stage, the Design Researcher asked both participant groups to reflect on their own experience of the outpatient department in response to probing questions from a topic guide developed by the two field researchers (the Design Researcher and Medical Sociologist) from looking at Appreciative Inquiry discovery interviews [21]. The questions centered around six stages of the outpatient journey: 1) preparation for appointment; 2) reception; 3) the waiting experience; 4) consultation; 5) leaving the appointment; 6) returning home and lasting memories. For staff participants, the questions dealt with their experience of working in the MS clinic: 1) preparing for clinic; 2) reception area; 3) interacting with patients while they wait; 4) consultation rooms; 6) the close of clinic. The metaphor of a journey was used, with maps designed to metaphorically navigate the outpatient service.

**Figure 2:**
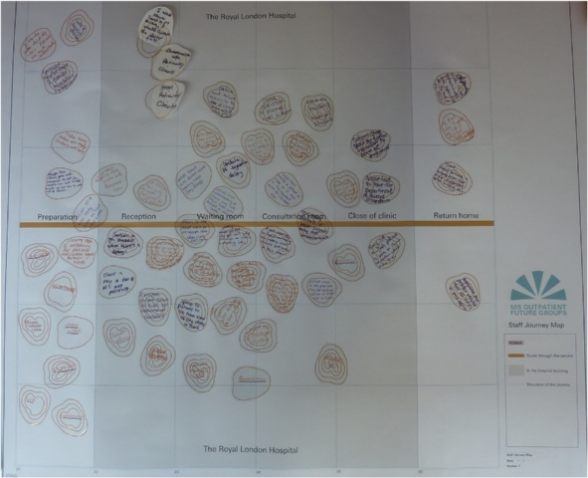
**Outpatient journey map complete with contours after discussing the staff future group, Stage 1.**

**Figure 3:**
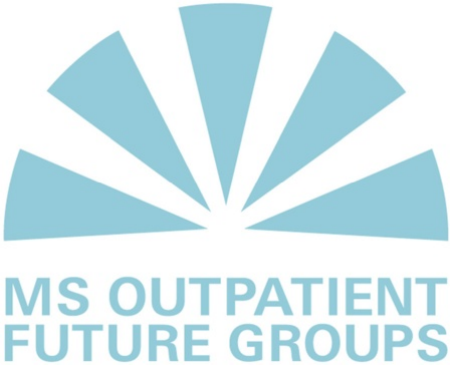
**The MS Outpatient Future Group logo.**

Each journey map was in the form of an information diagram, compiled from conversations with the Professor of Neurology and the Design Researcher’s observations of the clinic. People use maps in various forms to navigate the world so the visual identity of the study and the journey maps were designed to give the impression of an Ordnance Survey map (Figure 2) with a colour scheme and layout mimicking this, so that patients could immerse themselves in the analogy. In the maps, an orange coloured road relates to the individual’s journey through the outpatient department and blue grid lines represent the six stages of the journey’s structure. The journey starts on the left hand side of the map and travels horizontally through each of the six stages of the map to the right hand side, representing the start and the end of the outpatient journey. The study logo features the study title below the icon of a viewpoint taken from an Ordnance Survey map (Figure 3).

**Figure 4:**
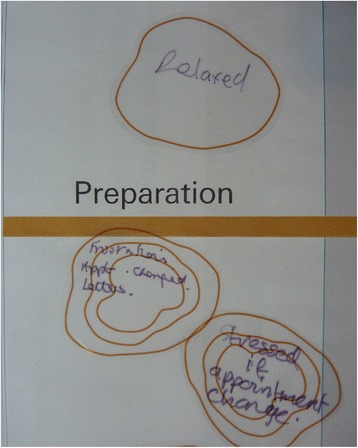
**Contour stickers with participants’ thoughts and feelings written on them around the ‘preparation’ stage of the patient outpatient journey.**

**Figure 5:**
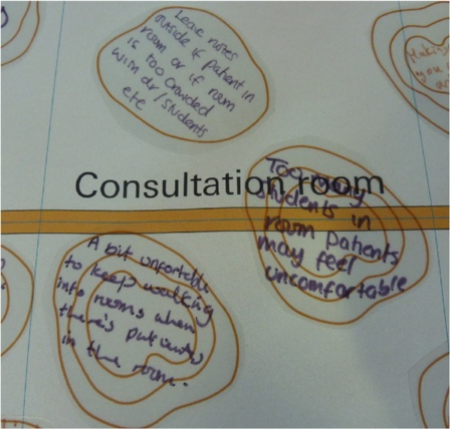
**Contour stickers with participants’ thoughts and feelings written on them around the ‘consultation room’ stage of the staff outpatient journey.**

Stickers that looked like the contours on a map were provided for the participants to represent their feelings (Figure 4 and 5). For example a one contour line sticker represented a calm feeling, whereas the more contours (traditionally meaning a hill), the more intense the feeling such as being scared, anxious or happy. When replying to the questions, participants were encouraged to write their feelings on a sticker and stick them onto the map at the relevant place. This visually linked the feelings of the participants to specific stages within the service. Over time the layers of contours built up, giving a 3-dimensional representation of the ‘landscape’ of their journey. The areas of intense feelings became immediately obvious.

### Stage 2 – combining maps and discussing an ideal journey

The second stage combined the groups of patients and staff. The Design Researcher encouraged discussion around the journey maps from stage one, considering each interaction from both staff and patient perspectives. Sharing the feelings on the maps allowed a group of participants to understand how the other group perceived the service and experience interactions.

The participants were then encouraged to use an analogy for communicating the nature of the lived experience [22]. The idea of going on an ideal (pleasurable) journey, was used as the analogy to best illustrate the different stages of a desired patient journey. The underpinning artistic approach served as a foundation to understand the participant’s world [23], and encouraged non-formulaic discussion.

**Figure 6:**
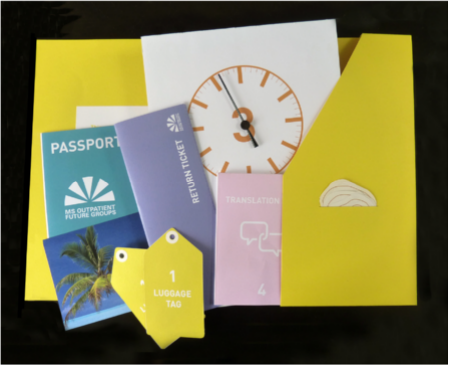
**Set of props given to each participant in Stage 2.**

**Figure 7:**
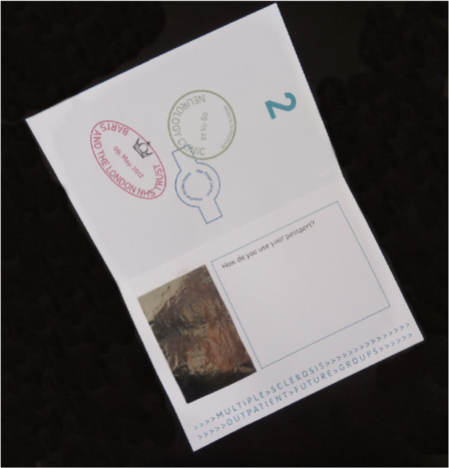
**The passport prop used by participants to imagine behaviours around using documents to travel to the next stage of a journey.**

**Figure 8:**
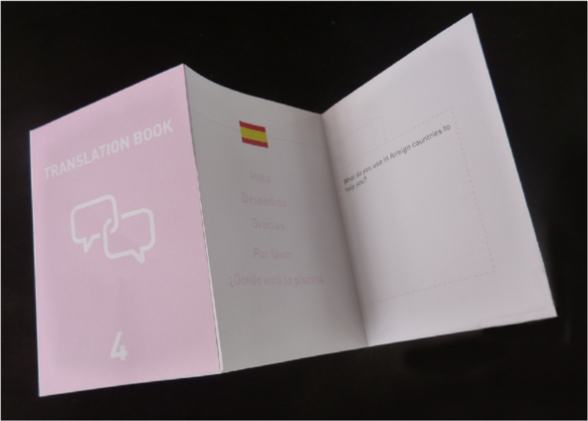
**The translation book prop used by participants to imagine behaviours around translating languages.**

Each participant was given a set of props to prompt thinking about the interactions they have throughout their ideal journey and their feelings associated with them (Figure 6). The props were used to encourage participants to engage in the analogy, record thinking and increase the interaction of participants in the session [24]. Each prop related to each of the six stages that was being discussed: 1) luggage tag: for preparation; 2) passport: for checking-in (Figure 7); 3) departure clock: for waiting; 4) book on translation: to use at the destination (Figure 8); 5) return ticket: for departure booking; 6) postcard: for lasting memories.

Again the group was asked open questions about their feelings, as in the first stage, to start the discussion, then the participants responded by writing the interaction that they would carry out on the paper prop in front of them. The Ideal Journey Map’s six stages were labelled similarly to the previous two journey maps but with titles relating to traveling on an ideal journey; for example ‘packing’ instead of ‘preparation’, ‘check-in’ instead of ‘reception’, and ‘destination’ instead of ‘consultation’. Participants were able to consider the behaviours around ideal interactions, for example how they research new locations they were travelling to, how they order from a foreign menu and how they plan to travel to the airport. At each of the six stages, the participants were able to suggest how their service interaction while travelling could become a future interaction in the outpatient service. This activity encouraged participants to suggest new interactions and improvements that could be provided for future patients and staff throughout the outpatient journey. This included receiving more information or interacting with staff in new ways, in order to achieve their ideal feelings.

At the end of the activity, one of the participants read out the comments on the props and allowed the rest of the group to cluster these as proposed improvement ideas. It was crucial that this came from the participants to enhance the interpretation and validity of research findings.

### Stage 3 – developing service improvements

**Figure 9:**
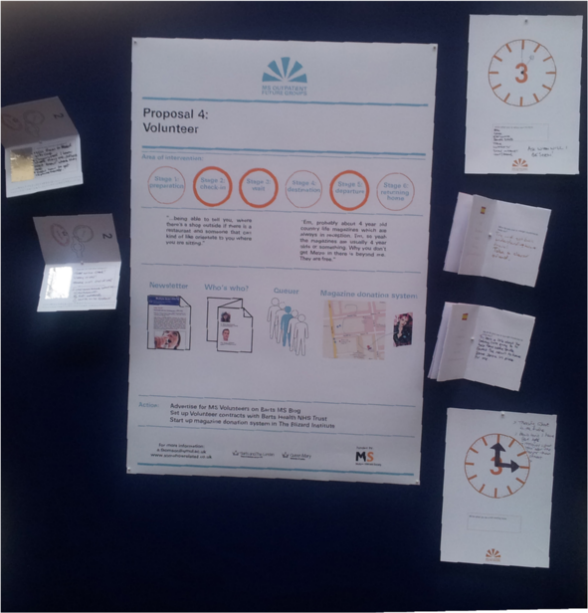
**Example of one presentation board with prototypes used to present proposals back to participants in Stage 3.**

In the third and final stage, the staff and patient participants met separately. The service improvement ideas were presented back to participants in the form of prototypes that were visualised and presented through a series of conference style posters that the Design Researcher developed (Figure 9). These were in keeping with the visual identity of the other resources in the project.

The aim of the final stage was to discuss how to implement the new service improvements in the clinic. Senior nursing staff management, the Barts Health NHS Trust volunteer service, an MS Specialist Nurse and the Professor of Neurology attended the staff group to endorse and strengthen the transition of the improvements into clinic procedure.

## Results

### Use of the analogy

Participants responded to using the analogy of an ideal journey in different ways. One patient participant, right at the outset of the activity, stated that they understood how the props and the analogy were to be used to think differently about the outpatient service.

*I suppose with all these things, as much the same way you go the doctors or to the hospital, I can see that there’s a good correlation between the two things. It’s quite good, it's a good way of doing it, looking at it from a slightly different angle.* (Patient Participant, Future group Stage 2).

Six participants shared their experience of travelling and three of these were able to make direct suggestions for service improvements. For example, one participant describes how she researches new locations before she travels then relates to how this would be useful behavior for the outpatient clinic.

*So I suppose what is there, slightly different is em, where, what is the destination, what the nice restaurants what is the nice places for the kids, still related to MS, but just beaches, woods or whatever, but em, in relation to hospital I think, there is never ever a map with em, or there is never ever a eh ‘drinks are over there,’ never, and I think that would be really useful.* (Patient Participant, Future group, Stage 2).

After the initial analogy was successfully adopted by the participants it then brought about a further seven analogies of service experiences. Three participants used other analogies of non-health related service experiences such as barbers, airports, travel companies and car services. Four participants referred to service experiences of other health clinics such as maternity, diabetes and cancer services. The example below demonstrates how staff describe the volunteer in the epilepsy clinic to the patient participants.

### Conversation exerpt

- Staff Participant A: *…you’ve got epilepsy patients and they have volunteers that come in and they have a stand with that information stand and the volunteer the patients can come and chat to. And they give them leaflets and things liked that.*
- Staff Participant B: *It’s in the coffee room, its in the photocopy room.*
- Patient Participant: *That’s usually a lay person isn’t it.*
- Staff Participant A: *Yeah it's usually a volunteers that can have epilepsy themselves so they are speaking about experience.*
- Patient Participant: *That’s perfect that's perfect, you know.*

This proposal was generated independently of the props and solely from the analogy. Participants went on to describe how the role of the clinic volunteer was much like that of a holiday rep’, welcoming passengers to the location.

Some patient participants found it difficult to engage with the analogy of thinking of their hospital visit as ‘ideal’ or like ‘going on a holiday’. Three participants did not refer to any analogy at all. One patient stated that she had not been on holiday in a long time so can't remember what it is like. One participant described how she felt initially the idea of ‘going on holiday’ to be ‘silly’ and found it hard to relate attending outpatients to going on holiday.

### Use of the props to suggest improvements

The participants spent time examining the props when they first received them and discussed them and their purpose. They were then used by participants throughout the sessions to make notes and think about the questions posed by the Design Researcher and the group discussion. All of the ideation in this stage was by the participants using the props in the discussion. For example, when the translation book was introduced to the group, participants discussed how they translate foreign languages and were able to relate this to the idea in health.

### Conversation exerpt

- Patient Participant A: *I learn a little bit of the language, yeah I do, just to orientate myself, learn the basics. Bonjour, Oui, you know the absolute basics to get me through. Knowing that I won’t have, I can’t master all of it. So I can understand some of the very simple communication, very simple, almost child like actually. You know, very quite basic.*
- Staff Participant C: *The basic, please, thank you.*
- Patient Participant C: *Yes because in your case of going to, France when you did it’s pretty alienating and you shut yourself away which is awful.*
- Staff Participant C: *Yeah yeah, I was completely ‘cos I was already in this country, two, just two years and I was scared me to death I didn't want to go there.*
- Patient Participant C: *It’s pretty alienating, in fact the experience, coming too close to medical jargon etc. can be really really alienating.*

When the passport was introduced to the group, discussions then revolved around patient notes. Staff were able to suggest how the maternity services deal with notes.

### Conversation exerpt

- Staff Participant C: *I’m straying from the subject now but…I see why they want to see their notes as they do it elsewhere like midwifery.*
- Staff Participant B: *Yes.*
- Patient Participant E: *Why is it like that in midwifery?*
- Staff Participant B: *Cause they can have a baby anywhere, that's why. That’s why, you have to carry.*
- Staff Participant C: *So the same format should be eh eh you know.*
- Patient Participant C: *Because following that theme you could become ill anywhere.*
- Staff Participant C: *Of course*
- Staff Participant B: *Yes. And also…this is why we get so many*.
- Staff Participant C: *If patient comes to hospital the first thing they should hand over is the passport, the notes!*

### Service improvements

The participants discussed what they would like for an ‘ideal’ service or what resources they would create for an ‘ideal’ experience. The Design Researcher then prototyped these ideas and they were then discussed in stage three. It was important that in stage three, each participant had the opportunity to comment on each of the prototypes and proposals. The improvements, listed below, have either been implemented or are being currently tested in the MS outpatient clinic.

1. Volunteer in clinic: The role of a volunteer in the Neurology clinic was very popular within the group. Following the study, one of the participants that took part in the study applied for the first volunteer role. This has now developed into a Patient Advisory Group which meets every three months to advise on the development of the clinic improvements, future research studies and contributed to the rebranding of the entire MS research team.
2. A guide to the MS clinic for new patients: This resource collates learnings from expert patients of the service for new patients. The information includes how to change your appointment, a description of professional roles in the clinic and how to contact staff alongside prompt questions to prepare patients for their appointment while waiting. A topic that arose regularly was that of ‘sharing knowledge’. This was between patients, sharing their experience and also between patients and staff. The idea of the clinic guide came from a patient comment about sharing information of a travel destination between friends. The group decided this could work well as a guide to the clinic for future patients who are new to the service. This is currently being evaluated in the outpatient clinic every week by the clinic volunteer. This feedback is then given to the Design Researcher who is iterating the design and the information.
3. MS Clinic Dictionary: This resource was developed from the ‘translation book’ prop discussions. It was decided that a dictionary would be useful for patients as participants felt that within consultations medical jargon was used frequently and there was still a lot of uncertainty about medical procedures. Many comments referred to searching for information on the internet and participants felt that having a reliable source, specifically dealing with the MS service at the RLH, would be incredibly valuable.
4. Walking map: The map displays walking distances from the closest underground station and bus stops to the outpatient clinic. Also located on the map are recommended coffee shops near the RLH, the closest cash machine and disabled parking bays. All distances were measured with a trundle wheel to ensure their accuracy. This initiative is being developed into a research study around how PwMS esitmate walking distances.
5. Magazine replenishment: Weekly the magazines are replenished in all of the outpatient clinics from the Research Insitute adjacent to the RLH.
6. Training: Informal training was delivered to update the outpatient staff on recent developments in MS research and care from the MS Specialist Nurses.

## Discussion

### Use of props

The physical props, in conjunction with the visual mapping, supported the use of the analogy and engaged participants to discuss possible future outpatient interactions. Participants were cognitively engaged by spending time choosing the contours, organising them on the relevant position on the map, making notes and relating to how they could use the props. This use of verbal and non-verbal platforms involved a variety of cognitive processes in making sense of the presented information, promoting a deeper learning [25]. There was a risk that the physical objects – the contours, the OS Maps and the props - could have overshadowed the discussion but as the group was large it worked well, as some participants took a lot of time thinking and writing on the props whereas others immediately responded when the props were distributed.

One participant specially commented on the attention to the detail and craft of the props, and on how the highly emotive contours made her feel that her ideas and thoughts were valued. These physical objects were designed to be similar to realistic objects that would be used by people while travelling to prompt the participants into thinking creatively. The high level of finish of the objects showed an effort to convey this which went far beyond current co-design tools, for example the temporary nature of post-it notes.

It is clear, with the example of the translation book, how the choice of prop influenced the discussions around medical jargon and the development of the MS Clinic Dictionary. The props sparked these discussions and the ideation that followed. Participants were easily able to continue discussing what should be included in this information resource and how it should be distributed within the clinic. The conversation then followed onto how it could be developed to cater for people who were without a definitive diagnosis of MS or people who would not want to know about the more degenerative stages of the disease. These more complex discussions and considerations could not have been reached without a deeper understanding of the cultural implications of developing information resources within this setting.

It is important to explain the reasoning behind the choice of analogy and props by the Design Researcher. Significant consideration was given to how the analogy could engage participants to share their experiences and provide a starting point for participants to imagine new interactions, engaging them in the speculation event. The props as objects were carefully designed to be interesting and easy to use. However, there were implications of the choice of analogy and props conditioning the types of ideas suggested for implementation. This did not disadvantage the potential of the ideation tools as the Design Researcher had a wider understanding of the time, cost and practical resources available to implement any suggested improvements and designed the props to reflect this.

### Use of speculation

The inclusion of the Design Researcher as the main facilitator explored the potential of this approach for generating new ideas in health service improvement activities. In this study it was not the Design Researcher’s role to design the service improvements, but to facilitate the participants’ creative processes and prototype and produce the ideas the participants generated, which were then reviewed in the final future group. The Design Researcher was able to encourage participants in their discussion, while also highlighting innovative ideas that had potential for implementation.

Sharing results with research participants in stage three acknowledged their contributions to the research study and was an essential component of knowledge transfer [26]. This feedback session was crucial in the way that the Design Researcher ensured that the staff could make sense of the new improvements and discuss what the changes would mean for them [27]. The presence of senior members of staff ensured the improvements would be implemented and especially for staff, their contributions to the project acknowledged. Discussions between participants and senior members of staff centered around the projected scenarios of staff and patient interactions, allowing participants to discuss the implications of the idea before its use.

### The use of design-led methods

The process used to create these service improvements could be described as a designerly way of doing things [12] in the way the method plays on the reality of the going on an ideal journey and prototyping ideas, yet this process depended on the ideas and skillset of the staff and the patients to suggest the proposals. The props were designed from the understanding that the Design Researcher had of the MS Clinic and what type of improvements would be feasible within the study’s scope. The props were able to engage the participants in imagining how new resources could be used before they existed.

Reflecting on how design-led methods were utilised in this study, the props and the behaviors they evoked adhered to the cultural setting in which they would be used. In this case, the MS outpatient clinic procedure was able to be likened to that of preparing and travelling on a journey, and the stages of both could be mirrored, highlighting the importance for adapting participatory methods to consider cultural attributes. These props, however, would possibly not be suitable for other clinics if the clinic procedure is different and the analogy irrelevant.

Employing the analogy of ‘travelling on an ideal journey’ evokes and makes certain ethical, economic and social assumptions. In order to address this and ensure the activities were as inclusive as possible, the props were designed with no reference to any specific travel company, mode of transportation or location of destination. When one participant mentioned they had not been on holiday in a long time when others were discussing their holiday experiences, they were encouraged to think about an ‘ideal journey’ of any form e.g. travelling to a local destination.

Although the analogy was used to draw potential similarities between an ideal journey and an ideal outpatient experience, there are inherent differences between these contexts of experience. For example, the role of someone seeking care as a patient is different to choosing to experience a service through traveling for leisure. This potentially challenging comparison did not arise in the study discussions, although participants did comment on how many of their suggestions would not cost anything to develop, which reflects an awareness of the limited resources of the NHS.

This brings us to consider the potential benefits and limitations for deploying methods from the design field within health service improvement activities. The aim of this is often considered to be the stimulation of creative thinking [5], yet the tools within approaches such as EBCD lack the ability to create innovative design proposals out of the group discussion stages [28]. This study has shown the potential for design-led methods to further explore the ‘ideation’ stage of service improvement activities through more subjectively designed tools. The props and the analogy facilitated conversations which explored the possibilities of what the service experience could be for each participant, which is closer to more explorative methods of design-led research [29]. The authors of this paper argue that this provided a better quality of interaction between participants, enabling them to not just suggest solutions to problems but create and share more radical suggestions for change (such as initiating a Patient Advisory Group). However, there is a danger that adapting design-led research methods to use in health service improvement activities may lose their creative nature through being applied in procedural manners to generate and gather data for analysis [30]. For example, cultural probes are inherently playful and ambiguous in nature and need to be re-designed and adapted to the cultural settings in which they are used, which has previously relied on the skills of a professional designer [28]. The challenge then becomes ensuring the creative qualities of these methods are not lost. We define quality here as valuing design-led methods’ inherent uncertainty while paying attention to the playful nature of how they are used. Regardless, adapting any design-led method in service improvement projects would require substantial work to prove its effectiveness to create long-term change in practice.

## Conclusion

This paper has described how the MS Outpatient Future Group used design-led methods to engage staff and patient participants in service improvement activities. The study built on the focus group method to create future groups which use speculation to inspire participants to imagine alternative health experiences. This demonstrates the potential for social science methods to integrate design-led approaches while not sacrificing the established principles of the method or the creative potential to generate successful ideas for service improvement.

This paper has also shown that in developing new ideation tools, there needs to be consideration of the final context of use, as with this study where there was a clear link between choice in methods of interaction and the service improvements that were developed. Therefore it would not be unrealistic to think that other props would have produced different proposals to improve the service.

The MS Outpatient Future Group study considered not only how participants would engage in the study but also interact with each other through the discussion activities. The physical tools, the language used to describe the process, the methods of participation all valued participants’ contributions, supporting them in being creative and guiding them through quite an unusual process.

## Abbreviations

EBCD: Experience Based Co-design
MS: Multiple Sclerosis
PWMS: People with Multiple Sclerosis
RLH: Royal London Hospital
